# Altered levels of circulating insulin-like growth factor I (IGF-I) following ischemic stroke are associated with outcome - a prospective observational study

**DOI:** 10.1186/s12883-018-1107-3

**Published:** 2018-08-06

**Authors:** N. David Åberg, Daniel Åberg, Katarina Jood, Michael Nilsson, Christian Blomstrand, H. Georg Kuhn, Johan Svensson, Christina Jern, Jörgen Isgaard

**Affiliations:** 1 Department of Internal Medicine, Institute of Medicine, The Sahlgrenska Academy at University of Gothenburg, Gröna Stråket 8, SE-413 45 Gothenburg, Sweden; 2 Department of Clinical Neuroscience, Institute of Neuroscience and Physiology, The Sahlgrenska Academy at University of Gothenburg, Gothenburg, Sweden; 3 Hunter Medical Research Institute, University of Newcastle, Newcastle, Australia; 4 Center for Stroke Research Berlin, Charité – Universitätsmedizin Berlin, Berlin, Germany; 5 Institute of Biomedicine, The Sahlgrenska Academy at University of Gothenburg, Gothenburg, Sweden; 6 Department of Clinical Genetics, The Sahlgrenska Academy at University of Gothenburg, Gothenburg, Sweden

**Keywords:** Insulin-like growth factor I, Ischemic stroke, Outcome

## Abstract

**Background:**

Insulin-like growth factor I (IGF-I) has neuroprotective effects in experimental ischemic stroke (IS). However, in patients who have suffered IS, various associations between the levels of serum IGF-I (s-IGF-I) and clinical outcome have been reported, probably reflecting differences in sampling time-points and follow-up periods. Since changes in the levels of post-stroke s-IGF-I have not been extensively explored, we investigated whether decreases in the levels of s-IGF-I between the acute time-point (median, 4 days) and 3 months (ΔIGF-I, further transformed into ΔIGF-I-quintiles, ΔIGF-I-q) are associated with IS severity and outcome.

**Methods:**

In the Sahlgrenska Academy Study on Ischemic Stroke (SAHLSIS) conducted in Gothenburg, Sweden, patients with IS who had s-IGF-I measurements available were included (*N* = 354; 65% males; mean age, 55 years). Baseline stroke severity was evaluated using the National Institutes of Health Stroke Scale (NIHSS) and converted into NIHSS-quintiles (NIHSS-q). Outcomes were assessed using the modified Rankin Scale (mRS) at 3 months and 2 years.

**Results:**

In general, the levels of s-IGF-I decreased (positive ΔIGF-I), except for those patients with the most severe NIHSS-q. After correction for sex and age, the 3rd ΔIGF-I-q showed the strongest association to mRS 0–2 [Odds Ratio (OR) 5.11, 95% confidence interval (CI) 2.18–11.9], and after 2 years, the 5th ΔIGF-I-q (OR 3.63, 95% CI 1.40–9.38) showed the strongest association to mRS 0–2. The associations remained significant after multivariate correction for diabetes, smoking, hypertension, and hyperlipidemia after 3 months, but were not significant (*p* = 0.057) after 2 years. The 3-month associations withstood additional correction for baseline stroke severity (*p* = 0.035), whereas the 2-year associations were further attenuated (*p* = 0.31).

**Conclusions:**

Changes in the levels of s-IGF-I are associated primarily with temporally near 3-month outcomes, while associations with long-term 2-year outcomes are weakened and attenuated by other factors. The significance of the change in post-stroke s-IGF-I is compatible with a positive role for IGF-I in IS recovery. However, the exact mechanisms are unknown and probably reflects combinations of multiple peripheral and central actions.

**Electronic supplementary material:**

The online version of this article (10.1186/s12883-018-1107-3) contains supplementary material, which is available to authorized users.

## Background

Extensive studies conducted in experimental animals have demonstrated that insulin-like growth factor-I (IGF-I) exerts neuroprotective and plasticity-promoting effects [1, 2]. For humans who have suffered an ischemic stroke (IS), a few observational studies have evaluated the role of endogenous levels of serum IGF-I (s-IGF-I). In the first two studies on this topic (*N* = 85 and *N* = 42, respectively), s-IGF-I was associated with measures of improved functional outcome [3, 4]. However, s-IGF-I was analyzed at only one time-point, either within 24 h of IS onset [3] or at 19–209 days after the IS [4], and functional follow-up was performed approximately 3–6 months post-stroke [3, 4]. In addition, a previous study from our group (*N* = 407) has shown a positive association between the 3-month level of s-IGF-I and improvements in the mRS score from 3 months to 2 years, whereas there was a negative association with the 3-month mRS score [5]. Thus, although endogenous s-IGF-I has been associated with favorable IS outcome, there remain unresolved issues in terms of the importance of the: post-stroke sampling time-point; age of the patient; severity of IS; timing of the follow-up; and temporal changes in s-IGF-I level after IS.

The association between change in post-stroke s-IGF-I and IS outcome has been investigated in one small study, in which the average level of s-IGF-I increased by 8.5% from < 72 h to 7 days after the stroke (*N* = 15) [6]. However, in that study, there was relatively large inter-individual variability, and interestingly, a decrease in post-stroke s-IGF-I correlated with a better1-month mRS score (*N* = 10) [6]. Furthermore, the local expression of IGF-I in the brain may increase after IS [7, 8], and in analogy with the increased brain uptake of IGF-I after experimental exercise [9], the transport of IGF-I from the serum into the brain may be increased after IS. In our previous study, the level of s-IGF-I was increased during the first days after the stroke (+ 11.2% on Days 0–2, as compared to healthy controls), followed by a leveling off of the s-IGF-I level on Days 3–5. Thereafter, there was an average decrease of 14.8% from Day 9 to Day 19, resulting in 3-month s-IGF-I levels that were approximately similar to those seen in healthy, age-matched controls [5]. However, we did not analyze the associations between the individual changes in s-IGF-I and IS outcome [5]. The hypothesis underlying the present study is that not only the absolute levels of s-IGF-I, but also the temporal pattern of s-IGF-I levels (as estimated by changes in the s-IGF-I levels), are of importance for IS outcome. Thus, our primary objective was to investigate whether intra-individual changes in post-stroke s-IGF-I (ΔIGF-I), from the acute phase to 3 months post-IS, are associated with functional independence 3 months after the IS, and if so, whether ΔIGF-I is also associated with functional outcome 2 years after IS. As ΔIGF-I has not been investigated extensively to date, we explored descriptively the effects of the following parameters: first day of sampling; age; IS severity; stroke subtype; and stroke etiology. We also performed multivariate regression analyses with the inclusion of potential confounders, such as cardiovascular risk factors and IS severity.

## Methods

### Subjects and methods

**Fig. 1:**
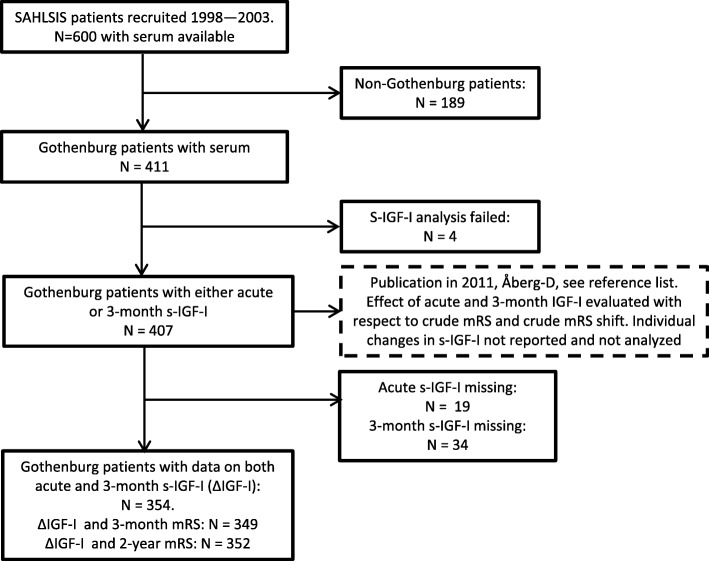
Flow chart showing numbers of included subjects and reasons for the exclusion of other patients

**Fig. 2:**
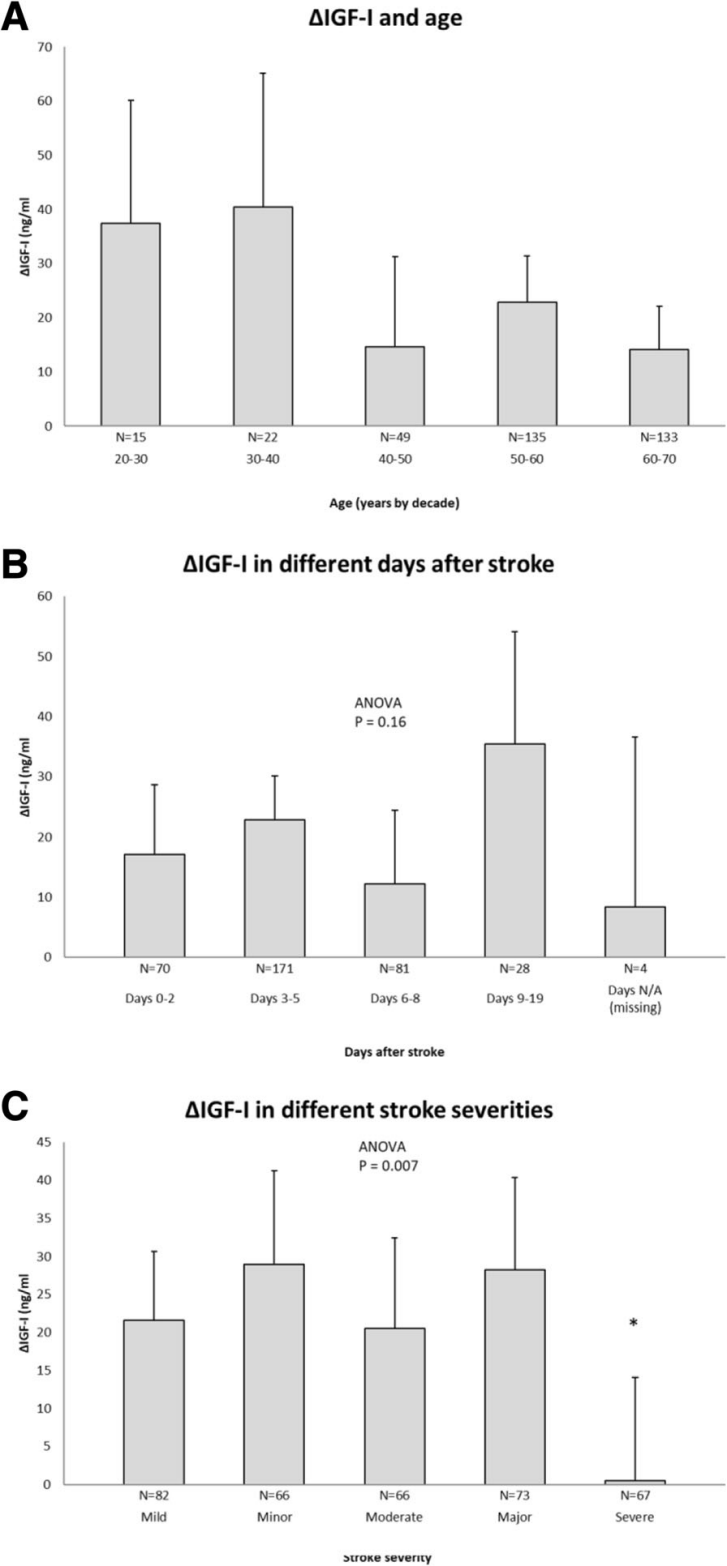
Descriptive data on ΔIGF-I in relation to age, sampling day, and ischemic stroke (IS) severity. The error bars are 95% confidence intervals (CI), and the numbers (N) of subjects in each category are shown. **a** ΔIGF-I for patients of different ages, as expressed by age decade at index IS. **b** ΔIGF-I values for different days post-IS. **c** ΔIGF-I values for the five IS severity levels of the National Institutes of Health Stroke Scale (NIHSS) (for limits and details, see *Methods* section). Significance levels were analyzed by ANOVA, followed by post-hoc Dunnett’s test with the first group as reference. If the ANOVA was non-significant no further testing was performed. *p < 0.05

The design of SAHLSIS has been reported elsewhere [10]. Briefly, patients (< 70 years of age) with first-ever or recurrent acute IS were recruited consecutively at four Stroke Units in western Sweden between 1998 and 2003 (see Fig. 1 for flow chart of inclusions). The final inclusion cohort with regard to ΔIGF-I had 354 subjects (Table 1). S-IGF-I was analyzed on one occasion in 2008 with a methodological intra-assay coefficient of variation (CV) of 5.1% and the biological variation showed a CV of 38% [5]. Blood sampling was performed between 08:30 and 10:30 after overnight fasting, and s-IGF-I was assayed using an IGF-binding protein (IGFBP)-blocked RIA kit (Mediagnost, Reutlingen, Germany). The acute serum samples were taken 0–19 days after the IS, with a median sampling time of 4 days [5]. The frequencies of previous hypertension, diabetes mellitus, and smoking were recorded and, the levels of low-density lipoprotein (LDL) were evaluated as previously described [10]. The ΔIGF-I values were transformed into quintiles (ΔIGF-I-q), owing to the values being skewed towards the left, as well as for convenience of presentation. The ΔIGF-I-q were defined according to ΔIGF-I: q1 = − 279.6--10, q2 = − 9.999–10, q3 = 10.001–30.7, q4 = 30.701–55.6, q5 = 55.601–178.9 ng/ml, with higher quintiles representing a decrease in s-IGF-I from the acute time-point to the 3-month time-point. Blood glucose or plasma glucose was analyzed using standardized methods at the Department of Clinical Chemistry at the Sahlgrenska University Hospital. In cases with the presence of blood glucose, these values were transformed to plasma glucose according to the formula: plasma glucose = blood glucose × 1.11. Initial stroke severity was assessed by scoring using the Scandinavian Stroke Scale (SSS), with the values being recalculated into the now more commonly used National Institutes of Health Stroke Scale (NIHSS). The algorithm used was: NIHSS = 25.68–0.43 × SSS [11], and due to a markedly skewed appearance, these scores were further transformed into quintiles: q1 = 0–0.74 (mild); q2 = 0.7401–2.03 (minor); q3 = 2.0301–3.75 (moderate); q4 = 3.75–10.2 (major); and q5 = 10.201–42 (severe). Due to many cases having minimal NIHSS scores, the first quintile was somewhat overbalanced (see Fig. 2). Stroke subtype and etiology were classified according to the Oxfordshire Community Stroke Project (OCSP) [12] and the Trial of Org 10,172 in Acute Stroke Treatment (TOAST) [13] criteria (see Table 2 for groups and abbreviations). Outcomes were assessed functionally and classified according to the modified Rankin Scale (mRS), where the levels of functional independence was classified by scores in the range of 0–2 (considered as favorable) and scores in the range of 3–6 (considered as unfavorable) [14]. Further details of the study design, patient examination, scoring scales, and protein measurements are given in the Additional file 1. A part of this report was presented as an abstract (with a modified title) at the European Stroke Conference in May 2018 [15] .

**Table 1:** Baseline data for patients and s-IGF-I in each of the quintiles of changing s-IGF-I (ΔIGF-I-q1–5)

Parameter		Unit		Value
n				354
Age at index ischemic stroke		Years (SD)		55.4 (11)
Sex		Missing (N)		229/125
		Male/female (fraction)		0.65
Diabetes		Yes (N/fraction)		67 (0.19)
		Missing (N)		0
Hypertension		Yes (N/fraction)		188 (0.53)
		Missing (N)		0
Current smoking		Yes (N/fraction)		136 (0.38)
		Missing (N)		0
LDL level (ng/nL)		Mean (SD)		3.3 (1.0)
		Missing (N)		27
P-glucose (acute)		Mean (SD)		6.5 (2.64)
		Missing (N)		8
P-glucose (3 m)		Mean (SD)		6.03 (2.29)
		Missing (N)		8
Stroke severity (NIHSS)		Mean (20, 80%)		5.3 (0.7, 10.2)
		Missing (N)		0
Stroke outcome (mRS) 3 m		Mean(SD)		1.85 (1.06)
		Missing (N)		5
Stroke outcome (mRS) 2 yr		mRS (SD)		1.77 (1.32)
		Missing (N)		2
Dead (3-24 m)		Yes (n/fraction)		9 (0.025)
		Missing (N)		0
s-IGF-I (acute)		ng/mL (SD)		172.8 (62.9)
		Missing (N)		0
s-IGF-I (3 m)		ng/mL (SD)		152.7 (55.7)
		Missing (N)		0
ΔIGF-I (ng/mL), all data		ng/mL (SD)		20.2 (51.0)
		Missing (N)		0
Quintile of ΔIGF-I:		ΔIGF-I (ng/mL)	s-IGF-I acute (ng/mL)	Change (%)
	ΔIGF-I-q1 (SD)	− 48.3 (48.0)	146.5 (53.7)	incr. 33.0
	(N)	71	71	71
	ΔIGF-I-q2 (SD)	1.6 (5.8)	145.4 (44.6)	decr. 1.10
	(N)	72	72	72
	ΔIGF-I-q3 (SD)	20.8 (5.7)	152.5 (56.0)	decr 13.5
	(N)	71	71	71
	ΔIGF-I-q4 (SD)	42.5 (7.2)	178.9 (45.9)	decr 23.6
	(N)	70	70	70
	ΔIGF-I-q5 (SD)	85.9 (28.0)	240.5 (57.6)	decr 35.7
	(N)	70	70	70

Absolute ΔIGF-I (ng/mL) represents a subtraction of acute s-IGF-I by 3-month s-IGF-I. A negative numerical value represents an increase from the acute to 3-month time point, and a positive numerical value represents a decrease. Modified Rankin scale (mRS), low density lipoprotein (LDL), National Institutes of Health Stroke Scale (NIHSS). An extended version of the table with a comparison to data presented in 2011 is found in Additional file 2: Table S1

**Table 2:** S-IGF-I for patients included by etiology, stroke subtype and severity, respectively

	Mean		Mean		Mean			
	ΔIGF-I	P	Acute IGF-I	P	3-month IGF-I	P	Age	P
	(SD)		(SD)		(SD)			
Stroke severity (NIHSS†)		Dunnett, q1 vs. q2-q5						
q1 (mild)	21.6 (42.0)	N/A	162.4 (59.5)	N/A	140.8 (50.0)	N/A	53.1 (12.5)	N/A
N	82		82		82		82	
q2 (minor)	29.0 (51.0)	ns	181.9 (67.0)	ns	152.9 (53.1)	ns	55.7 (9.9)	ns
N	66		66		66		66	
q3 (moderate)	20.6 (49.5)	ns	168.1 (63.5)	ns	147.6 (56.0)	ns	54.4 (11.1)	ns
N	66		66		66		66	
q4 (major)	28.3 (72.7)	ns	181.6 (61.8)	ns	153.4 (53.6)	ns	58.4 (9.5)	0.01
N	73		73		73		73	
q5 (severe)	0.58 (58.5)	0.04	171.7 (62.5)	ns	171.2 (63.2)	0.003	54.0 (10.7)	ns
N	67		67		67		67	
Missing (n)	0		0		0		0	
Subtype (OCSP)		Tukey’s crosswise						
Lacunar cerebral infarction (LACI)	21.1 (37.0)	0.09-TACI	163.9 (57.4)	ns	142.8 (48.5)	**TACI	56.6 (9.6)	0.05-POCI
N	113		113		113		113	
Partial anterior cerebral infarction (PACI)	18.1 (47.3)	ns	172.8 (67.9)	ns	154.7 (59.5)	ns	55.0 (11.3)	ns
N	104		104		104		104	
Posterior cerebral infarction (POCI)	30.7 (59.4)	**TACI	181.6 (65.4)	ns	150.9 (57.1)	ns	52.7 (12.2)	0.11-TACI
N	94		94		94		94	0.05-LACI
Total anterior cerebral infarction (TACI)	−1.5 (68.1)	**POCI	171.7 (58.4)	ns	173.3 (57.1)	**LACI	52.7 (9.3)	0.11-POCI
N	37	0.09-LACI	37		37		37	
Missing (N)	6		6		6		6	
Etiology (TOAST)		Tukey’s crosswise						
Large vessel diseaase (LVD)	20.4 (53.7)	ns	181.1 (57.0)	*D	160.8 (57.9)	ns	59.0 (7.8)	***D, **Cr
N	51		51	0.08-CE	51		51	
Small vessel diseaase (SVD)	19.1 (39.6)	ns	161.4 (60.6)	***D	142.3 (53.4)	**D	58.4 (7.2)	***D, **Cr
N	69		69		69		69	
Cardioembolic (CE)	6.1 (46.2)	ns	151.2 (53.3)	***D, 0.08-LVD	145.1 (51.1)	*D	55.5)11.6)	*D
N	52		52	0.15-Cr	52		52	
Cryptogenic (Cr)	20.1 (43.3)	ns	174.1 (60.7)	***D	154.0 (53.9)	0.09 vs. D	53.1 (11.9)	**LVD/SVD
N	112		112	0.15-CE	112		112	
Arterial dissection (D)	40.1 (95.7)	*CE	223.7 (63.0)	***SVD/CE/Cr	183.6 (66.7)	**-SVD	48.1 (9.4)	***LVD/SVD
N	27		27	*LVD	27	*CE, 0.09-Cr	27	**Cr, 0.14-D
Other/undeterrmined	26.2 (45.7)	N/A	172.2 (71.9)	N/A	146.0 (53.7)	N/A	54.2 (13.5)	N/A
n	43		43		43		43	
Missing (N)	0		0		0		0	

*P*-values less than 0.3 are specified although they are considered being not signicant (ns). * < 0.05, ** < 0.01. *** < 0.001 for Dunnett’s or Tukey’s post-hoc tests as indicated. As we have a slightly different number of included patients, and using NIHSS instead of SSS as it was presented in 2011, we present s-IGF-I (acute, 3-month) for comparison with the present inclusion (*n* = 354). TOAST = Trial of Org 10,172 in Acute Stroke Treatment [1], OCSP = Oxfordshire Community Stroke Project [4]. All variation shown is given in standard deviations (SD). N/A designates not applicable. †Scoring of neurological function is taken from the original SSS scores and converted to NIHSS (methods)

### Statistical analysis

Statistical evaluation was performed using the SPSS ver. 21.0 software (SPSS Inc., Chicago, IL). In the descriptive section, comparisons between groups (stroke severity, day-of-sampling, age, stroke subtype, and etiology) were performed using analysis of variance (ANOVA), and with Dunnett’s (for comparison with one reference) or Tukey’s (for crosswise comparison of all groups) post hoc tests, as indicated. Comparisons between distributions were made with the Chi-square test. Crude correlations using the method of Pearson are presented.

The aim of the project was to evaluate the effect of ΔIGF-I on functional outcome (mRS) 3 months and 2 years after IS. Towards this goal, we assessed whether the crude correlations withstood subsequent binary logistic regression analysis, in which the data on functional outcome were dichotomized (favorable outcome, mRS 0–2 versus unfavorable outcome, mRS 3–6). The Odds Ratios (ORs) and 95% confidence intervals (CIs) for favorable outcome (mRS 0–2) were age- and sex-adjusted (model 1) and were relative to the lowest quintile of ΔIGF-I (ΔIGF-I-q1, i.e., an increase in ΔIGF-I). Adjustments were also made for the vascular risk factors of smoking, hypertension, diabetes, and LDL levels (model 2), initial stroke severity quintile (model 3), and finally, also for the different days of first sample (model 4). As suggested by Peduzzi and coworkers [16], the number of events per variable (EPV) needed to reduce statistical bias should exceed the included number of covariates by a factor of 10. With the eight covariates given above, and an event rate of mRS 3–6 (*N* = 82 and *N* = 74 for the 3-month and 2-year outcomes, respectively), we refrained from adding ‘stroke etiology’ and ‘stroke subtype’ as covariates. The statistical significance level was set at *p* < 0.05.

## Results

### Descriptive data for s-IGF-I and stroke severity and subtype

The baseline characteristics of the 354 patients from SAHLSIS (Fig. 1) with ΔIGF-I values are summarized in Table 1. ΔIGF-I, which represents the intra-individual decrease in s-IGF-I from the acute phase to 3 months post-IS, averaged 20.2 ng/mL for the entire group. ΔIGF-I only weakly correlated with age (*r* = − 0.12, *p* = 0.025, *N* = 354), as compared to the acute s-IGF-I and age (*r* = − 0.331, *p* < 0.001, *N* = 354) and the 3-month s-IGF-I and age (*r* = − 0.264, *p* < 0.001, *N* = 354). Furthermore, the weak negative correlation between ΔIGF-I and age was not reflected in any difference of ΔIGF-I with respect to decade of age (Fig. 2a). We found no significant difference in ΔIGF-I regardless of the post-stroke sampling day of the first “acute” serum sample (Fig. 2b). However, ΔIGF-I was found to be related to initial stroke severity (Table 2, Fig. 2c). Specifically, there was no decrease in ΔIGF-I in the most severe IS, whereas the value of ΔIGF-I (20–29 ng/mL) was similar for all the other severities of IS. The low ΔIGF-I values noted for the patients with severe or large IS were further supported by the observed tendency towards a correlation between the ΔIGF-I quintiles and NIHSS quintiles (*r* = − 0.092, *p* = 0.084, *N* = 354), and the fact that the subtype with the largest IS, total anterior cerebral infarctions (TACI), had a lower ΔIGF-I than the other subtypes (Table 2, OCSP). Moreover, the ΔIGF-I value was higher in the group of patients with IS with etiology of arterial dissection, which might be attributable in part to the younger age of these patients (Table 2, TOAST classification). These subgroups were not used in the subsequent regressions, since the numbers of subjects and events in each of the groups were relatively low. As the absolute levels of s-IGF-I are inversely related to P-glucose levels and metabolic syndrome [17–19], ΔIGF-I could potentially relate to the P-glucose levels. However, there were no correlations (acute P-glucose and ΔIGF-I; *r* = − 0.065, *p* = 0.23, *N* = 346; 3-month P-glucose and ΔIGF-I, *r* = − 0.012, *p* = 0.83, *N* = 346), and this parameter was not used in the subsequent regression analyses.

### Multivariate regression analysis of changes in the levels of s-IGF-I and the clinical outcomes 3 months and 2 years after the IS

**Fig. 3:**
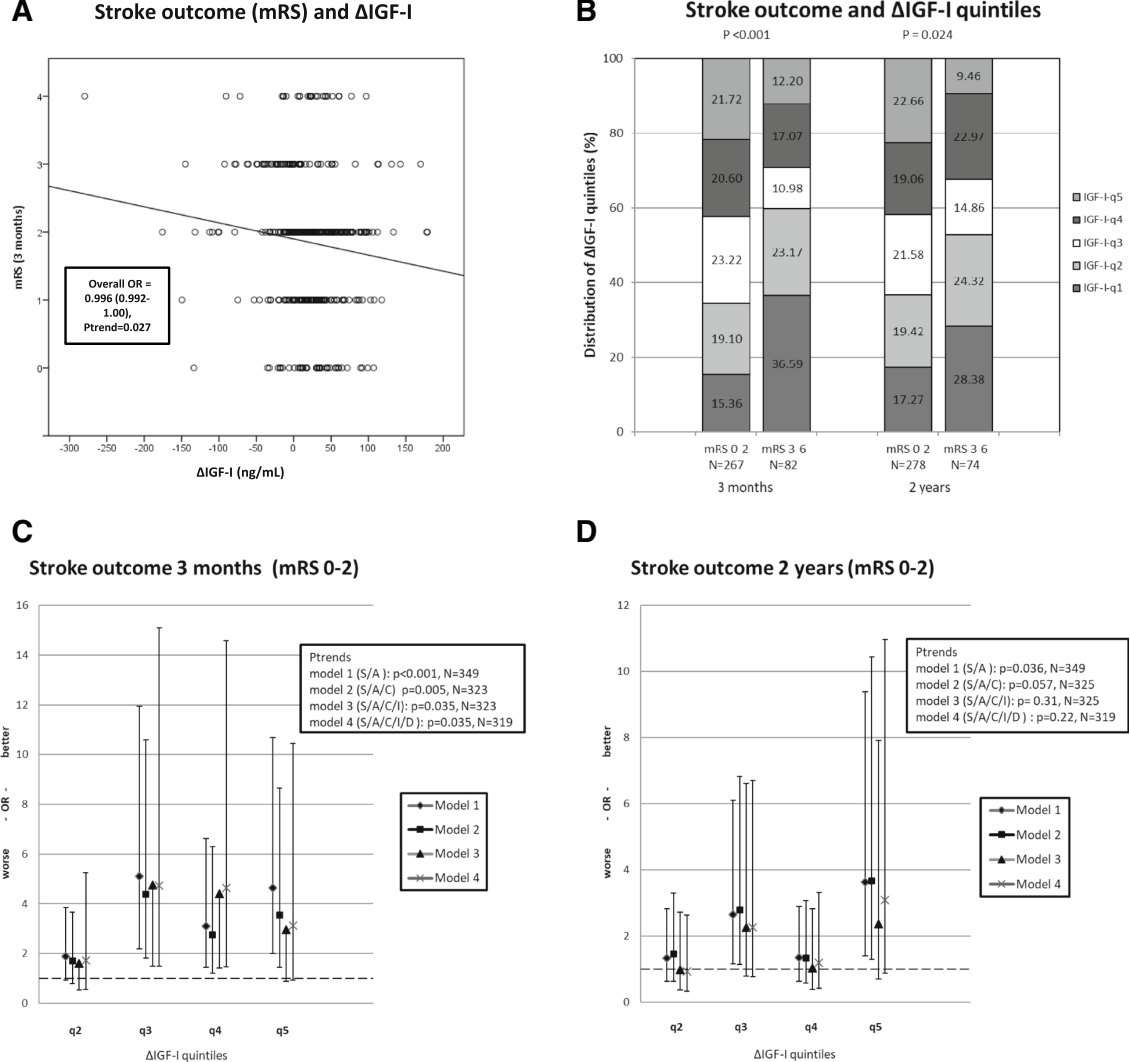
Stroke outcome in relation to ΔIGF-I. **a**. Distribution of crude ΔIGF-I values and crude 3-month mRS scores (*N* = 349). The box shows the overall Odds Ratios (OR) and 95% confidence intervals of an ordinal regression with mRS score as the dependent variable. For convenience, the line represents a crude correlation (*r* = − 0.114, *p* = 0.033). **b**. Unadjusted ΔIGF-I-quintile distribution (%) of stroke outcomes as indicated by mRS scores of 0–2 (good or favorable) or 3–6 (poor or unfavorable) 3 months and 2 years after IS. The *p*-values from the Chi-square analysis comparing distributions of good and poor outcome are shown. **c**. Functional outcome 3 months post-IS, shown as OR and 95% CI for associations (binary logistic regression) of favorable mRS score with unfavorable functional outcome for each of the ΔIGF-I quintiles relative to ΔIGF-I q1 (q1 is a reference with OR = 1, shown as a hatched line). Models 1–4 are shown with successively added adjustments for sex (S), age (A), traditional cardiovascular covariates (C), initial stroke severity (I), and day of the first blood sample (D), together with their respective numbers (N) with complete datasets. The boxes show the p-values for the overall associations using ΔIGF-I quintiles as a continuous variable with the same respective adjustments (p-trends). **d**. Functional outcomes 2 years after IS shown as OR and 95% CI for associations (binary logistic regression) between favorable mRS score and unfavorable functional outcome for each of the ΔIGF-I quintiles, as in B

We investigated whether ΔIGF-I was related to IS outcome after 3 months and 2 years, using univariate (correlations) and multivariate regression analyses. Although ΔIGF-I correlated with the crude 3-month mRS score (Fig. 3a), outliers and the skewing of ΔIGF-I indicated that the ΔIGF-I quintiles were better suited to analysis of association, and the application of the widely accepted dichotomized mRS. Accordingly, the crude ΔIGF-I quintiles correlated with favorable outcome (i.e., mRS 0–2) 3 months after IS (*r* = 0.21, *p* = 0.01, *N* = 349) and 2 years after IS (*r* = 0.14, *p* = 0.007, *N* = 352). This is evidenced by the significantly different distributions of ΔIGF-I quintiles at both 3 months and 2 years for the patients with favorable outcome (mRS 0–2) and those with unfavorable outcome (mRS 3–6) of IS (Fig. 2b).

For the significant crude associations with mRS outcome 3 months and 2 years after the IS, we performed further analyses using binary logistic regression with adjustment for multiple covariates. Higher ΔIGF-I quintiles (representing decreased levels of s-IGF-I) adjusted for sex and age were indeed associated with favorable outcome in terms of mRS score after 3 months (model 1, for ΔIGF-I-q5, OR 4.63, 95% CI 2.01–10.7; Fig. 3c). This was not due to the higher level of acute s-IGF-I found in ΔIGF-I-q4–5 (model 1 with acute s-IGF-I as an additional covariate; data not shown). We did not explore the impact of acute s-IGF-I on outcome any further, as acute s-IGF-I has been previously demonstrated to be negatively correlated with outcome [5]. Furthermore, the 3-month associations withstood adjustment for traditional cardiovascular risk factors and adjustment for initial stroke severity (Fig. 3c, model 3). However, the associations were marginally more robust for ΔIGF-I-q3 than for ΔIGF-I-q5 (model 1, ΔIGF-I-q3: OR 5.11, 95% CI 2.18–11.9). Although ‘day of first sample’ did not crudely relate with ΔIGF-I (Fig. 3b), there was a biological rationale for including this parameter as a covariate (due to the initial increase in s-IGF-I; see *Introduction*), which marginally amplified the associations (Fig. 3, c and d, model 4). This was also reflected in the regression analysis in which samples collected on Days 0–2 (*N* = 70) were excluded, generating an inclusion cohort of *N* = 275. This regression analysis generated marginally higher ORs for favorable outcome (*N* = 275, model 1, ΔIGF-I-q3: OR 5.59, 95% CI 2.03–14.4).

In general, the associations were somewhat weaker for the 2-year outcome (model 1, ΔIGF-I-q5: OR 3.63, 95% CI 1.40–9.38). The association with favorable 2-year outcome persisted as a trend (*p* = 0.057) after adjustment for cardiovascular risk factors but was significant for specific ΔIGF-I-quintiles (model 2, ΔIGF-I-q5: OR 3.66, 95% CI 1.29–10.4). However, additional adjustment for initial stroke severity reduced associations to non-significant levels (*p* = 0.31). For the 2-year outcome, the association was most robust for ΔIGF-I-q5.

## Discussion

### Decreased level of IGF-I is associated with favorable outcome after ischemic stroke

This study investigated individual s-IGF-I changes (ΔIGF-I) from the subacute phase after IS to 3-month follow-up. In addition, we related the ΔIGF-I to outcome for up to 2 years after IS. The ΔIGF-I did not differ with respect to stroke severity, except that in the most severe stroke cases, there was only a minimal change in the levels of s-IGF-I. In general, the individual ΔIGF-I values showed that s-IGF-I decreased from the subacute phase to 3 months post-IS. There were more prominent decreases in the levels of s-IGF-I in patients with favorable outcome than in those with unfavorable outcome. A subsequent regression analysis of ΔIGF-I-q revealed robust associations with favorable outcome at 3 months and somewhat less-pronounced associations at 2 years after IS. These associations withstood adjustments for cardiovascular covariates in the 3-month and 2-year follow-ups. However, the associations withstood additional adjustment for initial stroke severity only in terms of the 3-month follow-up. Taken together, our data show that a dynamic decrease in the level of s-IGF-I from the subacute phase to 3 months post-stroke is strongly associated with better stroke outcome at 3 months, whereas the association with outcome at 2 years is weaker.

### Decreasing the level of IGF-I is associated with favorable outcome, also after correction for confounders

The effect of the decrease in the level of IGF-I might be confounded by various factors, such as stroke volume, initial stroke severity, higher initial s-IGF-I, and cardiovascular factors. While the exact stroke volume was not available, the TOAST classification gave some indication of the stroke volume [13]. Accordingly, large IS (TACI) and severe IS, as opposed to other groups, exhibited a minimal ΔIGF-I (i.e., unchanged s-IGF-I). Therefore, it could be argued that the worst IS scenario, with low ΔIGF-I, could explain the statistical associations. However, even within the group of most severe IS, similar ORs for favorable outcome were noted (data not shown). Furthermore, when adding ‘initial stroke severity’ as a covariate in the statistical analyses, the association between ΔIGF-I and favorable 3-month outcome remained, whereas the association between ΔIGF-I and favorable 2-year outcome was weakened to statistically non-significant levels. Thus, even after correction for initial stroke severity, the association between ΔIGF-I and favorable 3-month functional outcome persisted.

In the multivariate regression analyses, the association between ΔIGF-I and IS outcome was not confounded by the level of acute s-IGF-I. This suggests that the association between ΔIGF-I and favorable outcome is independent of the acute level of IGF-I. Given the previous report on the absolute levels of IGF-I [5], we did not explore further the effects of the acute or 3-month levels of IGF-I.

The beneficial effect of ΔIGF-I was essentially unchanged by the applied adjustments for cardiovascular covariates (Fig. 3b and c). This is of importance, as the absolute levels of s-IGF-I are lower in cases of metabolic syndrome and diabetes [17–19]. In summary, the change in s-IGF-I level appears to be an independent predictor of favorable outcome 3 months after IS, as this association withstands corrections for cardiovascular covariates, absolute levels of acute IGF-I, and initial stroke severity.

### Possible underlying mechanisms

In the present study, it was not possible to determine whether the changes in s-IGF-I reflect similar changes in the local availability of IGF-I in the brain. However, in experimental IS, IGF-I was upregulated early after IS, both locally [7, 8, 20] and in the serum [8], allowing for a subsequent decrease in the level of IGF-I. In our previous study, the levels of s-IGF-I increased during the first days after the stroke. The levels of s-IGF-I reached a plateau on Days 3–5, and thereafter, an average decrease of 14.8% was seen on Days 9–19, resulting in 3-month s-IGF-I levels that were approximately similar to those in healthy, age-matched controls [5]. It can be speculated that the higher levels of IGF-I around the brain injuries result in better recovery, and that after 3 months, the expression of IGF-I decreases if the injury exhibits recovery (implying that IGF-I is no longer needed). In line with this, brain injuries with little recovery would continue to have unchanged or even increased levels of IGF-I in the brain, and possibly also in the serum, after 3 months. If so, local IGF-I in the brain would not be an exacerbating agent, but instead a substance that could improve the clinical outcome of IS. From experimental studies, there is evidence that local astrocyte IGF-I expression mediates neuroprotection [21] and that locally delivered astrocyte IGF-I improves experimental stroke outcomes [22]. While the exact mechanisms are not known, they probably involve neuroprotection, as well as angiogenesis, neurogenesis, and neuronal sprouting (for reviews, see [1, 2]). Another possible explanation is that the patients with IS who exhibit a substantial recovery and favorable outcome are those with the greatest potential for transporting IGF-I from the serum into the brain [9]. Such a mechanism would give results similar to ours, although this notion is partly contradicted by our previous report that large infarctions have relatively higher absolute levels of both acute and 3-month s-IGF-I [5]. It should be pointed out that interactions between local brain IGF-I synthesis, circulating s-IGF-I (including peripheral sources and regulation from liver and bone [23]), and uptake of s-IGF-I through the blood-brain barrier are biologically plausible but very complicated to study and poorly understood in humans. A more stringent time series of IGF-I measurements in the serum and cerebrospinal fluid (CSF) in relation to IS might give some indication of the relative importance of the different sources of IGF-I.

### Changes in s-IGF-I levels in relation to previous studies and significance of favorable clinical outcome

Our main finding is that ΔIGF-I is associated with favorable outcome both 3 months and 2 years post-IS, although the association with functional outcome after 2 years loses significance after adjustment for initial stroke severity. In a previous smaller study (*N* = 15), a decrease in IGF-I level during the first week of stroke was associated with shorter length of stay, greater independence at 1 month (mRS), and discharging to home vs. remaining as an inpatient [6]. As compared to the study of Mattlage and coworkers, our study has a wider range of first days of sampling and a considerably later time-point for the second sample (3 months). While the variation of the first day of sampling is a weakness in the present study, we have partly addressed this problem by correcting for this parameter in the multivariate regression analysis (Fig. 3, c and d, model 4) and by excluding Days 0–2, resulting in somewhat stronger associations between ΔIGF-I and favorable functional outcome (mRS 0–2). Thus, the combined results of the present and the previous studies clearly suggest that a post-stroke decrease in the level of s-IGF-I is associated with improved clinical outcome after IS.

In the present study, the largest beneficial effects (ORs) observed for ΔIGF-I were in ΔIGF-I-q3 (for 3-month outcomes) and in ΔIGF-I-q5 (for 2-year outcomes), which correspond to decreases in s-IGF-I of 13.5 and 35.7%, respectively (Table 1). These relatively substantial changes in s-IGF-I support the notion that s-IGF-I plays a role in stroke pathophysiology and rehabilitation. In terms of the crude unadjusted data, 42.3% of the patients who showed an increase in s-IGF-I (ΔIGF-I-q1) had an unfavorable 3-month functional outcome, as compared to 12.7–20.3% of those patients who showed a decrease in s-IGF-I (q3 - q5 of ΔIGF-I). These are rather large differences, as an unfavorable outcome (mRS 3–6) means that the patient is requires assistance with daily living activities. Furthermore, the differences in associations noted between q3 and q5 for ΔIGF-I- and mRS 0–2 are overall rather small. Our most important finding is that decreases in s-IGF-I levels contrast with no change or an increase in the post-stroke level of s-IGF-I with respect to outcome. However, this needs to be evaluated in greater detail in larger clinical studies.

### Strengths and limitations

The methodological strengths of this study include consecutive recruitment of well-characterized patients with IS. Another advantage is the high hospitalization rate (84–95%) for stroke patients in Sweden [24], which has among the highest rates in Europe [25]. The relatively young age of the participants in the present study (mean age, 55 years), as compared to the mean age of all patients with IS in Sweden (approximately 76 years [26]), facilitated follow-up, with very few drop-outs and few cases of fatality, although it somewhat disfavored the inclusion of the bulk of cases of IS etiology, i.e., IS due to cardiovascular causes. In addition, the inclusion of patients of young age favored the inclusion of less-severe cases of IS. The fact that few patients were lost to follow-up makes other selection biases unlikely. The fact that the patients were recruited between 1998 and 2003 means that very few patients received thrombolysis (local arterial, *N* = 4, intravenous, *N* = 0) and that more of the patients received previous treatment with warfarin (*N* = 41) than is currently the case. Another drawback of this study is that there is no analysis of exact stroke volumes, although baseline stroke severity can be used as a marker of stroke lesion volumes with correlation coefficients of 0.62–0.64 [27, 28]. In addition, we chose to include only those patients for whom there was a complete dataset for both the subacute and 3-month s-IGF-I, giving 354 subjects, as compared to the 407 subjects in our previous report [5]. We do not believe that this introduced any systematic bias, given that the cardiovascular covariates, acute and 3-month s-IGF-I levels, and the 3-month and 2-year mRS scores were comparable to those previously reported [5] (see also the Additional file 2: Table S1). Other weaknesses include the relatively small sample size and the lack of replication in a different geographic area.

## Conclusions

Decreasing levels of s-IGF-I show clear associations with favorable outcome at 3 months and 2 years after IS, suggesting that the dynamics of IGF-I regulation is of importance, independent of the actual s-IGF-I levels. After adjustment for initial stroke severity, the 3-month association remained statistically significant, whereas the 2-year association lost significance. Thus, the changes in s-IGF-I levels are associated primarily with the temporally close (3-month) outcomes, while the associations with long-term (2-year) outcomes are weakened and attenuated by other factors. The post-stroke changes in the levels of s-IGF-I are compatible with a positive role for IGF-I in IS recovery, although the exact mechanisms are uncertain and probably reflect certain combinations of different factors. Exploration of the causality warrants further studies involving intra-individual serial analyses of IGF-I levels in the serum and CSF of patients who have suffered an IS.

## Additional files

Additional file 1: Supporting information. (DOCX 44 kb)

Additional file 2: **Table S1.** Baseline data as compared to the study in 2011 [5] (DOCX 33 kb)

## Abbreviations

CI: 95% confidence interval
IGF-I: Insulin-like growth factor I
IS: Ischemic stroke
mRS: Modified Rankin Scale
NIHSS: National Institutes of Health Stroke Scale
OCSP: Oxfordshire Community Stroke Project
SD: Standard deviation
SSS: Scandinavian Stroke Scale
TOAST: Trial of Org 10,172 in Acute Stroke Treatment

## References

[1] Åberg ND, Brywe KG, Isgaard J (2006). Aspects of growth hormone and insulin-like growth factor-I related to neuroprotection, regeneration, and functional plasticity in the adult brain. TheScientificWorldJournal.

[2] Sohrabji F, Williams M (2013). Stroke neuroprotection: oestrogen and insulin-like growth factor-1 interactions and the role of microglia. J Neuroendocrinol.

[3] Denti L, Annoni V, Cattadori E, Salvagnini MA, Visioli S, Merli MF, Corradi F, Ceresini G, Valenti G, Hoffman AR (2004). Insulin-like growth factor 1 as a predictor of ischemic stroke outcome in the elderly. Am J Med.

[4] Bondanelli M, Ambrosio MR, Onofri A, Bergonzoni A, Lavezzi S, Zatelli MC, Valle D, Basaglia N, Degli Uberti EC (2006). Predictive value of circulating insulin-like growth factor I levels in ischemic stroke outcome. J Clin Endocrinol Metab.

[5] Åberg D, Jood K, Blomstrand C, Jern C, Nilsson M, Isgaard J, Åberg ND (2011). Serum IGF-I levels correlate to improvement of functional outcome after ischemic stroke. J Clin Endocrinol Metab.

[6] Mattlage AE, Rippee MA, Sandt J, Billinger SA (2016). Decrease in insulin-like growth Factor-1 and insulin-like growth Factor-1 ratio in the first week of stroke is related to positive outcomes. J Stroke Cerebrovasc Dis.

[7] Beilharz EJ, Russo VC, Butler G, Baker NL, Connor B, Sirimanne ES, Dragunow M, Werther GA, Gluckman PD, Williams CE (1998). Co-ordinated and cellular specific induction of the components of the IGF/IGFBP axis in the rat brain following hypoxic-ischemic injury. Brain Res Mol Brain Res.

[8] Wang J, Tang Y, Zhang W, Zhao H, Wang R, Yan Y, Xu L, Li P (2013). Insulin-like growth factor-1 secreted by brain microvascular endothelial cells attenuates neuron injury upon ischemia. FEBS J.

[9] Carro E, Nunez A, Busiguina S, Torres-Aleman I (2000). Circulating insulin-like growth factor I mediates effects of exercise on the brain. J Neurosci.

[10] Jood K, Ladenvall C, Rosengren A, Blomstrand C, Jern C: Family history in ischemic stroke before 70 years of age: the Sahlgrenska Academy study on ischemic stroke. Stroke 2005, 36(7):1383–1387. 10.1161/01.STR.0000169944.46025.09 15933254

[11] Ali K, Cheek E, Sills S, Crome P, Roffe C (2007). Development of a conversion factor to facilitate comparison of National Institute of Health Stroke Scale scores with Scandinavian Stroke Scale scores. Cerebrovasc Dis.

[12] Bamford J, Sandercock P, Dennis M, Burn J, Warlow C (1991). Classification and natural history of clinically identifiable subtypes of cerebral infarction. Lancet.

[13] Adams HP, Bendixen BH, Kappelle LJ, Biller J, Love BB, Gordon DL, Marsh EE (1993). Classification of subtype of acute ischemic stroke. Definitions for use in a multicenter clinical trial. TOAST. Trial of org 10172 in acute stroke treatment. Stroke.

[14] Banks JL, Marotta CA (2007). Outcomes validity and reliability of the modified Rankin scale: implications for stroke clinical trials: a literature review and synthesis. Stroke.

[15] Åberg ND, Åberg D, Jood K, Nilsson M, Blomstrand C, Kuhn HG, Svensson J, Jern C, Isgaard J (2018). The change in circulating insulin-like growth factor I (IGF-I) after ischemic stroke is independently associated with outcome. European Stroke Organisation Conference: 2018.

[16] Peduzzi P, Concato J, Kemper E, Holford TR, Feinstein AR (1996). A simulation study of the number of events per variable in logistic regression analysis. J Clin Epidemiol.

[17] Landin-Wilhelmsen K, Wilhelmsen L, Lappas G, Rosen T, Lindstedt G, Lundberg PA, Bengtsson BA (1994). Serum insulin-like growth factor I in a random population sample of men and women: relation to age, sex, smoking habits, coffee consumption and physical activity, blood pressure and concentrations of plasma lipids, fibrinogen, parathyroid hormone and osteocalcin. Clin Endocrinol.

[18] Parekh N, Roberts CB, Vadiveloo M, Puvananayagam T, Albu JB, Lu-Yao GL (2010). Lifestyle, anthropometric, and obesity-related physiologic determinants of insulin-like growth factor-1 in the third National Health and nutrition examination survey (1988-1994). Ann Epidemiol.

[19] Spartano NL, Stevenson MD, Xanthakis V, Larson MG, Andersson C, Murabito JM, Vasan RS (2017). Associations of objective physical activity with insulin sensitivity and circulating adipokine profile: the Framingham heart study. Clinical obesity.

[20] Gustafson K, Hagberg H, Bengtsson BÅ, Brantsing C, Isgaard J (1999). Possible protective role of growth hormone in hypoxia-ischemia in neonatal rats. Pediatr Res.

[21] Genis L, Davila D, Fernandez S, Pozo-Rodrigalvarez A, Martinez-Murillo R, Torres-Aleman I (2014). Astrocytes require insulin-like growth factor I to protect neurons against oxidative injury. F1000Research.

[22] Okoreeh AK, Bake S, Sohrabji F (2017). Astrocyte-specific insulin-like growth factor-1 gene transfer in aging female rats improves stroke outcomes. Glia.

[23] Sjögren K, Liu JL, Blad K, Skrtic S, Vidal O, Wallenius V, LeRoith D, Törnell J, Isaksson OG, Jansson JO (1999). Liver-derived insulin-like growth factor I (IGF-I) is the principal source of IGF-I in blood but is not required for postnatal body growth in mice. Proc Natl Acad Sci U S A.

[24] Hallström B, Jonsson AC, Nerbrand C, Petersen B, Norrving B, Lindgren A (2007). Lund stroke register: hospitalization pattern and yield of different screening methods for first-ever stroke. Acta Neurol Scand.

[25] Malmivaara A, Meretoja A, Peltola M, Numerato D, Heijink R, Engelfriet P, Wild SH, Belicza E, Bereczki D, Medin E (2015). Comparing ischaemic stroke in six European countries. The EuroHOPE register study. Eur J Neurol.

[26] Asplund K, Hulter Asberg K, Appelros P, Bjarne D, Eriksson M, Johansson A, Jonsson F, Norrving B, Stegmayr B, Terent A (2011). The Riks-stroke story: building a sustainable national register for quality assessment of stroke care. Int J Stroke.

[27] Effect of intravenous recombinant tissue plasminogen activator on ischemic stroke lesion size measured by computed tomography. NINDS; the National Institute of Neurological Disorders and Stroke (NINDS) rt-PA stroke study group. Stroke 2000, 31(12):2912–2919. 10.1161/01.str.31.12.2912 11108748

[28] Menezes NM, Ay H, Wang Zhu M, Lopez CJ, Singhal AB, Karonen JO, Aronen HJ, Liu Y, Nuutinen J, Koroshetz WJ (2007). The real estate factor: quantifying the impact of infarct location on stroke severity. Stroke.

