# Supplementary material for: Altered levels of circulating insulin-like growth factor I (IGF-I) following ischemic stroke are associated with outcome - a prospective observational study
Source: BMC Neurol. 2018 Aug 6;18:106. doi: 10.1186/s12883-018-1107-3 (PMC6091156; doi:10.1186/s12883-018-1107-3)
Supplement: Supplementary file 1 — Supporting information. (DOCX 44 kb) [file 12883_2018_1107_MOESM1_ESM.docx]

**Altered levels of circulating insulin-like growth factor I (IGF-I) following ischemic stroke are associated with outcome - a prospective observational study**

N. David Åberg, Daniel Åberg, Katarina Jood, Michael Nilsson, Christian Blomstrand, H. Georg Kuhn, Johan Svensson, Christina Jern, Jörgen Isgaard.

**Additional file 1 (online only):**

**Additional supporting information:**

**Detailed description of study populations and clinical examination (item number)**

1. The Sahlgrenska Academy Study on Ischemic Stroke (SAHLSIS): The study population comprised Caucasian patients (n=844) who presented with first-ever (n=732) or recurrent (n=112) ischemic stroke (IS) before reaching the age of 70 years. The patients were consecutively recruited between 1998 and 2003 at four stroke units in Western Sweden. It can be mentioned that in a continuation of the study, which is not included here, patients have been included until 2008. Caucasian population controls (n=668) were included as previously reported [[1](#_ENREF_1), [2](#_ENREF_2)] for the 1998-2003 cohort. However, the total cohort was only accessible for genetic analysis, whereas fewer subjects were available with serum samples and complete clinical data (serum collected 1998-2003, n=600 patients and controls, respectively). In the present study, no data from controls were used. From the 600 patients, s-IGF-I levels were available from a previous study in 407 patients recruited at the Gothenburg hospital [[3](#_ENREF_3)]. S-IGF-I, either acute or at 3-months were missing in some cases, and as the present study focused on changes in s-IGF-I (from acute to 3-month s-IGF-I, ΔIGF-I) that were previously not investigated; there were 354 patients available with complete records on s-IGF-I.
2. Clinical definitions and descriptions: Ischemic stroke (IS) was defined as an episode of focal neurological deficits with acute onset and lasting >24 hours or until death, with no apparent non-vascular cause, and no signs of primary hemorrhage on brain imaging. All patients were examined by a physician trained in stroke medicine. Information on the subjects’ vascular risk factors was collected as described previously [[1](#_ENREF_1)]. Hypertension was defined by pharmacological treatment for hypertension, systolic blood pressure ≥ 160 mm Hg, and/or diastolic blood pressure ≥ 90mm Hg. Diabetes mellitus was defined by diet or pharmacological treatment, fasting plasma glucose ≥ 7.0 mmol/L, or fasting blood glucose ≥ 6.1 mmol/L. Smoking habit was coded as current versus never or former. All cases underwent ECG and neuroimaging with computed tomography (CT) and/or magnetic resonance imaging (MRI). Extracranial carotid and vertebral duplex ultrasound, MR angiography, catheter angiogram, transcranial Doppler ultrasound, transthoracic and/or transesophageal echo-cardiography were performed when clinically indicated.
3. Among cases, blood was collected within 10 days (median 4 days, range 1-15 days) of the index stroke event and at 3-month follow-up (median 101, range 85–125 days).
4. For the 354 included patients, maximum stroke severity within the first 10 days after the stroke was scored using the Scandinavian Stroke Scale (SSS). SSS is a 58-point scale, in which a higher score represents better functionality. The SSS is highly (but inversely) correlated to the National Institutes of Health Stroke Scale (NIHSS). In this paper, global SSS scores were transformed to NIHSS scores using a conversion algorithm [[4](#_ENREF_4)], further transformed into quintiles (see present paper for details).
5. Functional outcome 3 and 24 months after IS was assessed according to the modified Rankin Scale (mRS) [[5](#_ENREF_5)], later modified to seven steps (0-6) [[6](#_ENREF_6)]. The mRS score was dichotomized for favorable outcome (mRS 0-2) versus unfavorable outcome (death or dependency; mRS 3-6, for discussion, see [[6](#_ENREF_6)]).
6. The localization and extent of the ischemic stroke according to the Oxfordshire Community Stroke Project (OCSP) classification as lacunar cerebral infarctions LACI), partial anterior cerebral infarctions (PACI), posterior cerebral infarctions (POCI), and total anterior cerebral infarctions (TACI) [[7](#_ENREF_7)]. Stroke etiology was classified using the Trial of Org 10172 in Acute Stroke Treatment (TOAST) criteria [[8](#_ENREF_8)]. These were large vessel disease (LVD), small vessel disease (SVD, cardioembolic (CE), cryptogenic (Cr), arterial dissection (D), and other causes to the stroke (as also found in Table 2).
7. Blood sampling was performed between 0830 and 1030 h after overnight fasting. Serum was isolated within 2 h by centrifugation at 2000g at 4℃ for 20 min and stored at -80℃ for 5–10 years before assay. Serum was assayed for IGF-I with an IGF-binding protein (IGFBP)-blocked RIA using a commercial kit (Mediagnost, Reutlingen, Germany) [[3](#_ENREF_3)]. The samples were assayed as follows: all acute samples on one occasion, all 3-month samples on one occasion, and the control samples on one occasion. Because s-IGF-I is considered to be very stable [[9](#_ENREF_9)], we did not specifically test long-term stability in our study. In our samples, the intra-assay coefficient of variation (CV%) was on average 5.1%, and the biological variation showed a CV of 38% [[3](#_ENREF_3)].
8. All participants provided informed consent prior to enrolment. For participants who were unable to communicate, consent was obtained from their next-of-kin. This study was approved by the Ethics Committee of the University of Gothenburg.

**References:**

1. Jood K, Ladenvall C, Rosengren A, Blomstrand C, Jern C: Family history in ischemic stroke before 70 years of age: the Sahlgrenska Academy Study on Ischemic Stroke. Stroke 2005, 36(7):1383-1387.

2. Olsson S, Jood K, Blomstrand C, Jern C: Genetic variation on chromosome 9p21 shows association with the ischaemic stroke subtype large-vessel disease in a Swedish sample aged </= 70. European journal of neurology 2011, 18(2):365-367.

3. Åberg D, Jood K, Blomstrand C, Jern C, Nilsson M, Isgaard J, Åberg ND: Serum IGF-I levels correlate to improvement of functional outcome after ischemic stroke. The Journal of clinical endocrinology and metabolism 2011, 96(7):E1055-1064.

4. Ali K, Cheek E, Sills S, Crome P, Roffe C: Development of a conversion factor to facilitate comparison of National Institute of Health Stroke Scale scores with Scandinavian Stroke Scale scores. Cerebrovascular diseases (Basel, Switzerland) 2007, 24(6):509-515.

5. Rankin J: Cerebral vascular accidents in patients over the age of 60. II. Prognosis. Scottish medical journal 1957, 2(5):200-215.

6. Banks JL, Marotta CA: Outcomes validity and reliability of the modified Rankin scale: implications for stroke clinical trials: a literature review and synthesis. Stroke 2007, 38(3):1091-1096.

7. Bamford J, Sandercock P, Dennis M, Burn J, Warlow C: Classification and natural history of clinically identifiable subtypes of cerebral infarction. Lancet 1991, 337(8756):1521-1526.

8. Adams HP, Jr., Bendixen BH, Kappelle LJ, Biller J, Love BB, Gordon DL, Marsh EE, 3rd: Classification of subtype of acute ischemic stroke. Definitions for use in a multicenter clinical trial. TOAST. Trial of Org 10172 in Acute Stroke Treatment. Stroke 1993, 24(1):35-41.

9. Burns C, Rigsby P, Moore M, Rafferty B: The First International Standard For Insulin-like Growth Factor-1 (IGF-1) for immunoassay: preparation and calibration in an international collaborative study. Growth hormone & IGF research : official journal of the Growth Hormone Research Society and the International IGF Research Society 2009, 19(5):457-462.
