# Supplementary material for: Altered levels of circulating insulin-like growth factor I (IGF-I) following ischemic stroke are associated with outcome - a prospective observational study
Source: BMC Neurol. 2018 Aug 6;18:106. doi: 10.1186/s12883-018-1107-3 (PMC6091156; doi:10.1186/s12883-018-1107-3)
Supplement: Supplementary file 2 — Table S1. Baseline data as compared to the study in 2011 [5] (DOCX 33 kb) [file 12883_2018_1107_MOESM2_ESM.docx]

**Altered levels of circulating insulin-like growth factor I (IGF-I) following ischemic stroke are associated with outcome - a prospective observational study**

N. David Åberg, Daniel Åberg, Katarina Jood, Michael Nilsson, Christian Blomstrand, H. Georg Kuhn, Johan Svensson, Christina Jern, Jörgen Isgaard.

**Additional Table S1 (online only):**

**Additional supporting Table S1**

**Title:** Baseline data as compared to the study in 2011 [[1](#_ENREF_1)].

**Footnotes:** The column “present value” is for patients with complete ΔIGF-I (Table 1 in main report) and includes subjects with either acute or 3-month s-IGF-I, whereas the “Åberg-2011” column includes data from that study [[1](#_ENREF_1)]. Absolute ΔIGF-I (ng/mL) represents a subtraction of acute s-IGF-I by 3-month s-IGF-I (negative numerical values represent an increase in s-IGF-I). Modified Rankin scale (mRS), low density lipoprotein (LDL), National Institutes of Health Stroke Scale (NIHSS), *Scandinavian Stroke Scale (SSS) used in 2011, recalculated to NIHSS here. #mRS has in a few cases been re-evaluated in the course of the study, and therefore the values are marginally different from 2011 [[1](#_ENREF_1)]. The reason for the higher number of missing observations in 2011, is primarily because that study included all observations with either acute or 3-month s-IGF-I. The present study included observations with both acute and 3-month s-IGF-I (=ΔIGF-I) which had a more complete record in general. N/A, not applicable. **Reference:**

1. Åberg D, Jood K, Blomstrand C, Jern C, Nilsson M, Isgaard J, Åberg ND: Serum IGF-I levels correlate to improvement of functional outcome after ischemic stroke. The Journal of clinical endocrinology and metabolism 2011, 96(7):E1055-1064.
